# Dissociated Roles of the Inferior Frontal Gyrus and Superior Temporal Sulcus in Audiovisual Processing: Top-Down and Bottom-Up Mismatch Detection

**DOI:** 10.1371/journal.pone.0122580

**Published:** 2015-03-30

**Authors:** Takeshi Uno, Kensuke Kawai, Katsuyuki Sakai, Toshihiro Wakebe, Takuya Ibaraki, Naoto Kunii, Takeshi Matsuo, Nobuhito Saito

**Affiliations:** 1 Department of Neurosurgery, Graduate School of Medicine, The University of Tokyo, Bunkyo-ku, Tokyo, Japan; 2 Department of Cognitive Neuroscience, Graduate School of Medicine, The University of Tokyo, Bunkyo-ku, Tokyo, Japan; UCLA, UNITED STATES

## Abstract

Visual inputs can distort auditory perception, and accurate auditory processing requires the ability to detect and ignore visual input that is simultaneous and incongruent with auditory information. However, the neural basis of this auditory selection from audiovisual information is unknown, whereas integration process of audiovisual inputs is intensively researched. Here, we tested the hypothesis that the inferior frontal gyrus (IFG) and superior temporal sulcus (STS) are involved in top-down and bottom-up processing, respectively, of target auditory information from audiovisual inputs. We recorded high gamma activity (HGA), which is associated with neuronal firing in local brain regions, using electrocorticography while patients with epilepsy judged the syllable spoken by a voice while looking at a voice-congruent or -incongruent lip movement from the speaker. The STS exhibited stronger HGA if the patient was presented with information of large audiovisual incongruence than of small incongruence, especially if the auditory information was correctly identified. On the other hand, the IFG exhibited stronger HGA in trials with small audiovisual incongruence when patients correctly perceived the auditory information than when patients incorrectly perceived the auditory information due to the mismatched visual information. These results indicate that the IFG and STS have dissociated roles in selective auditory processing, and suggest that the neural basis of selective auditory processing changes dynamically in accordance with the degree of incongruity between auditory and visual information.

## Introduction

Accurate processing of auditory information supports various aspects of human life, ranging from survival to social communication. Within the brain, auditory information is not processed independently of input from other sensory organs, and auditory processing can be distorted by visual information [1–7]. For example, people misperceive the spoken syllable “pa” as “ta” if the auditory information is accompanied by visual input in which the speaker produces the lip movement “ka” [8]. This phenomenon, known as the McGurk effect, demonstrates that auditory information is automatically integrated with visual input even if the input is discrepant from the auditory information [9,10]. Accurate processing of auditory information therefore requires the ability to detect and ignore visual information that is incongruent with the auditory input, to prevent inappropriate audiovisual integration.

We hypothesized that this ability is supported by two dissociated brain regions. Studies using a unimodal visual stimulus have shown that a target of high saliency (i.e., difference from its neighboring distractors) is processed mainly in the sensory cortex in a bottom-up manner, whereas a non-salient distracter-resembling target is processed in a top-down manner with involvement of the higher cortex [11–13]. The discrepancy of a target to distractors is thus thought to be a primary determinant of the brain regions that are involved in processing the target. In selective processing of auditory information from audiovisual inputs, the syllable spoken by a voice is more detectable if it has larger mismatch with the syllable predicted from the lip movement. Thus, target-distractor discrepancy can be defined in audiovisual processing as the degree of mismatch between actual and visually predicted sounds, which suggests that the functional separation among brain region holds true for selective auditory processing. Specifically, bottom-up processing is executed to process target auditory information when the target has a large discrepancy to the auditory information predicted from visual input, and top-down processing is executed to process target auditory information when the target has small discrepancy to the predicted auditory information. Audiovisual mismatch has been reported to induce activity in the inferior frontal gyrus (IFG), especially Brodmann areas 44 and 45, and a posterior part of the superior temporal sulcus (STS) [14–17], and studies on unimodal auditory processing have suggested that salient and non-salient auditory targets are processed in the superior temporal and inferior frontal regions, respectively [18,19]. Given these findings, the IFG and STS should be respectively engaged in top-down and bottom-up processing of target auditory information from audiovisual inputs.

However, studies to date on audiovisual processing have focused on how auditory and visual information are integrated into a single perception and have not investigated the effects of target-distractor discrepancy on involved brain areas. In addition, findings on top-down versus bottom-up processing have been obtained mainly from single modality research. It is thus unknown whether the neural basis of audiovisual processing changes depending on the discrepancy between auditory information and visual information and whether the IFG and the STS play dissociated roles in this processing. In the present study, we manipulated the degree of audiovisual incongruence and investigated brain activity while subjects selectively processed auditory information from audiovisual inputs. For this investigation, we used electrocorticography (ECoG). ECoG accurately detects high gamma activity (HGA), which is strongly associated with neuronal firing [20] and reflects local brain activity [21–27] with a high spatial resolution. The information provided by ECoG can be used to elucidate the roles of the IFG and STS in selectively processing auditory information from audiovisual inputs and to further understand the neural basis of accurate auditory processing.

## Material and Methods

### Informed consent

This study was approved by the research ethics committee of the faculty of medicine at the University of Tokyo (approval number 1797). Written informed consent was obtained from each patient before testing.

### Subjects

Eight consecutive patients with intractable epilepsy underwent subdural electrode implantation over a wide region of the left lateral surface of the brain for diagnostic purposes at the University of Tokyo Hospital between November 2012 and December 2013. Two patients were excluded because of prolonged status epilepticus or a low intelligence quotient score on the Wechsler Adult Intelligence Scale-III (< 65). We studied the remaining six patients (mean age 27.0 years, range 20–37 years; three females). All patients were right-handed, and had normal or corrected-to-normal vision, normal hearing, and no known history of neurological disease except for epilepsy. The Wada test revealed that all patients had left language dominance (Table 1).

**Table 1:** Demographic and clinical characteristics of the six patients.

**Patient**	**Age, y /Sex**	**Epilepsy focus**	**Language dominant side**	**VIQ**	**Electrodes, n**
1	37/F	Bilateral temporal lobe	Left	73	173
2	26/F	Left MTL	Left	92	180
3	20/M	Left occipital lobe	Left	71	179
4	24/F	Left MTL	Left	65	179
5	25/M	Left MTL	Left	99	164
6	30/M	Left STG	Left	104	179

VIQ = verbal intelligence quotient on the Wechsler Adult Intelligence Scale-III, MTL = medial temporal lobe, STG = superior temporal gyrus.

### Behavioral task

**Fig 1:**
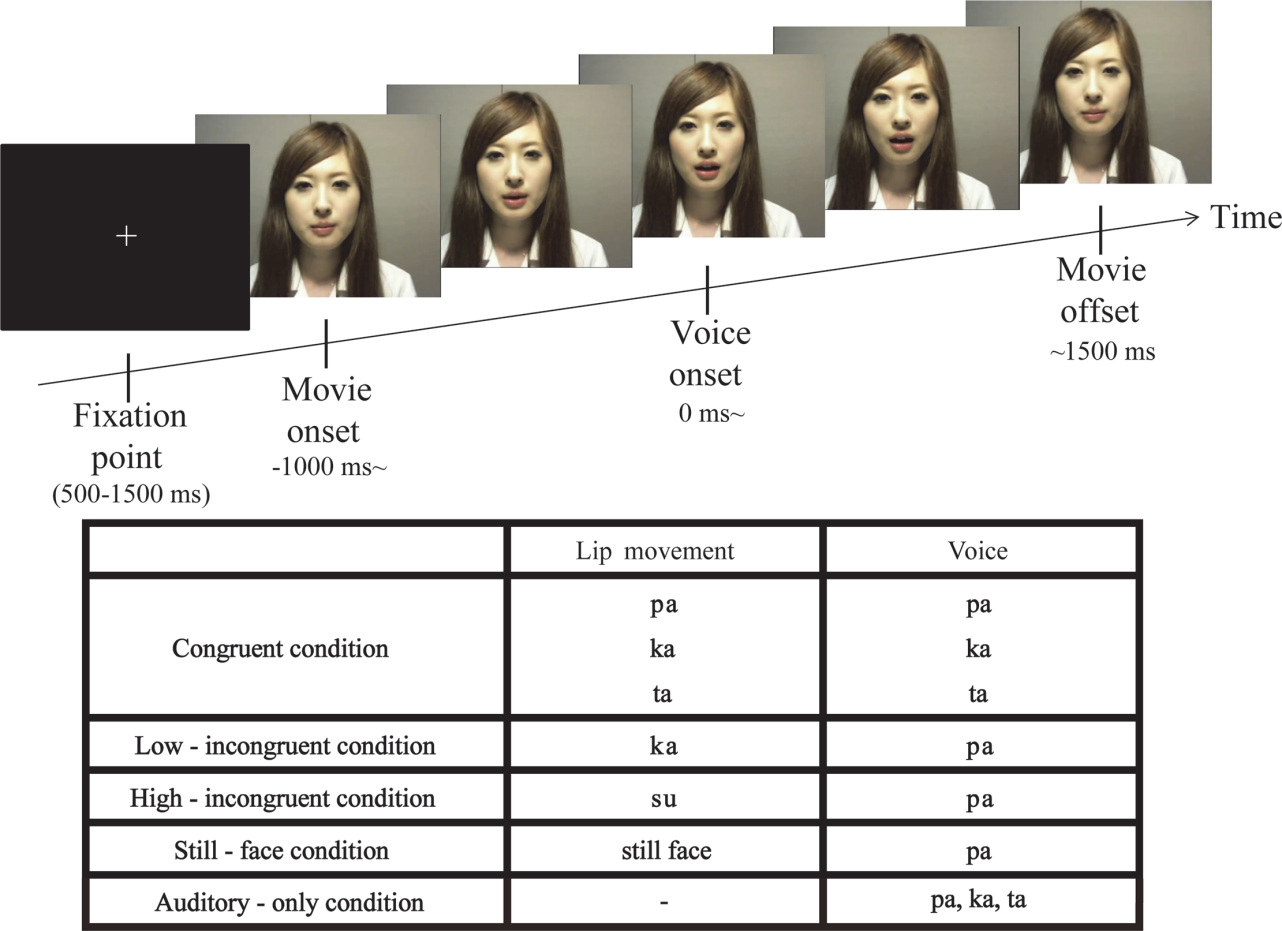
Experimental details. Patients were shown movie clips in which a Japanese female produced a lip movement of “pa,” “ka,” “ta,” or “su” with a voice speaking the syllable of “pa,” “ka,” or “ta.” In the congruent condition, the syllable spoken by the voice was congruent with the lip movement. In the incongruent conditions, the audiovisual information was mismatched: In the low-incongruent condition the voice “pa” was presented with the lip movement “ka” and in the high-incongruent condition the voice “pa” was presented with the lip movement “su”. A fixation point was presented for between 500 and 1500 ms before the onset of the movie clip. The interval between the onset of the movie clip and the onset of the audio was 1000 ms. The total length of each movie clip was 2500 ms.

We prepared movie clips of 2500 ms length to work in the Psychophysics Toolbox in MATLAB (The Mathworks Inc., Natick, MA, USA). In the movie clips, a Japanese female produced a lip movement of “pa,” “ka,” “ta,” or “su” while her voice spoke the syllable “pa,” “ka,” or “ta.” Based on the combination of the lip movement and the voice, there were four conditions: congruent, low-incongruent, high-incongruent, and still face (Fig 1). The individual in this manuscript has given written informed consent (as outlined in PLOS consent form) to publish these case details.

In the congruent condition, the auditory stimulus (i.e., the voice speaking the syllable “pa,” “ka,” and “ta”) was always matched with the visual stimulus (i.e., the speaker’s lip movement). In the two incongruent conditions, the audiovisual information was mismatched with different degrees of discrepancy: the speaker made a lip movement of “ka” and “su” in the low- and high-incongruent conditions respectively, and the voice speaking the syllable “pa” was presented in both conditions. The syllables “pa” and “ka” are plosive consonant and unrounded vowels, unlike the syllable “su”, which is a fricative consonant and rounded vowel [28,29]. Compared with the lip movement “ka” (i.e., the low-incongruent condition), the lip movement “su” (i.e., the high-incongruent condition) is more discrepant from the actual sound of the “pa.” To evaluate the different degrees of discrepancy, we conducted a pilot test for healthy subjects (mean age 29.9 years, range 27–34 years; two females; S1 Fig). The healthy subjects’ performance was worse in the low-incongruent than high-incongruent condition, supporting our assumption that the high-incongruent condition should have larger discrepancy. This performance profile was similar with those of patients in this study. In the still-face condition, the mouth of the female speaker remained closed while the voice “pa” was presented. This condition was included to ensure that the patient kept their attention on the visual stimulus, and the trials in this condition were not included in any analysis.

After watching each movie clip, patients were asked to report the syllable they heard by pressing a corresponding key. The choices were “pa,” “ka,” “ta,” or “no lip movement.” Thus, the correct answer was always “pa” in the low- and high-incongruent conditions, where the voice speaking the syllable “pa” was presented. In the still-face condition, the patient was asked to answer “no lip movement,” which guaranteed that the subject looked at lip movement of the speaker. Patients were not told that the lip movements and the voice of the speaker could be incongruent.

The congruent, low-incongruent, high-incongruent, and still-face conditions were conducted in a randomized order with an occurrence ratio of 0.45, 0.30, 0.15, and 0.1, respectively. The total number of these trials ranged from 66 to 264, depending on each patient’s condition.

The movie clips were 640 × 480 pixels and shown at a rate of 30 frames per second on a monitor placed about 70 cm in front of the patient. Sound was delivered at an intensity of approximately 75 dB through earphones that were digitalized at 44.1 kHz. The onset of the voice was 1 s after the onset of the movie clip. The onset of mouth movement was 270 to 570 ms before the onset of the voice, reflecting a natural articulation in recording. The total length of all clips was 2500 ms. The interval between clips ranged from 500 to 1500 ms. For the experiment, the patient was seated on a chair in an electrically shielded room.

**Fig 2:**
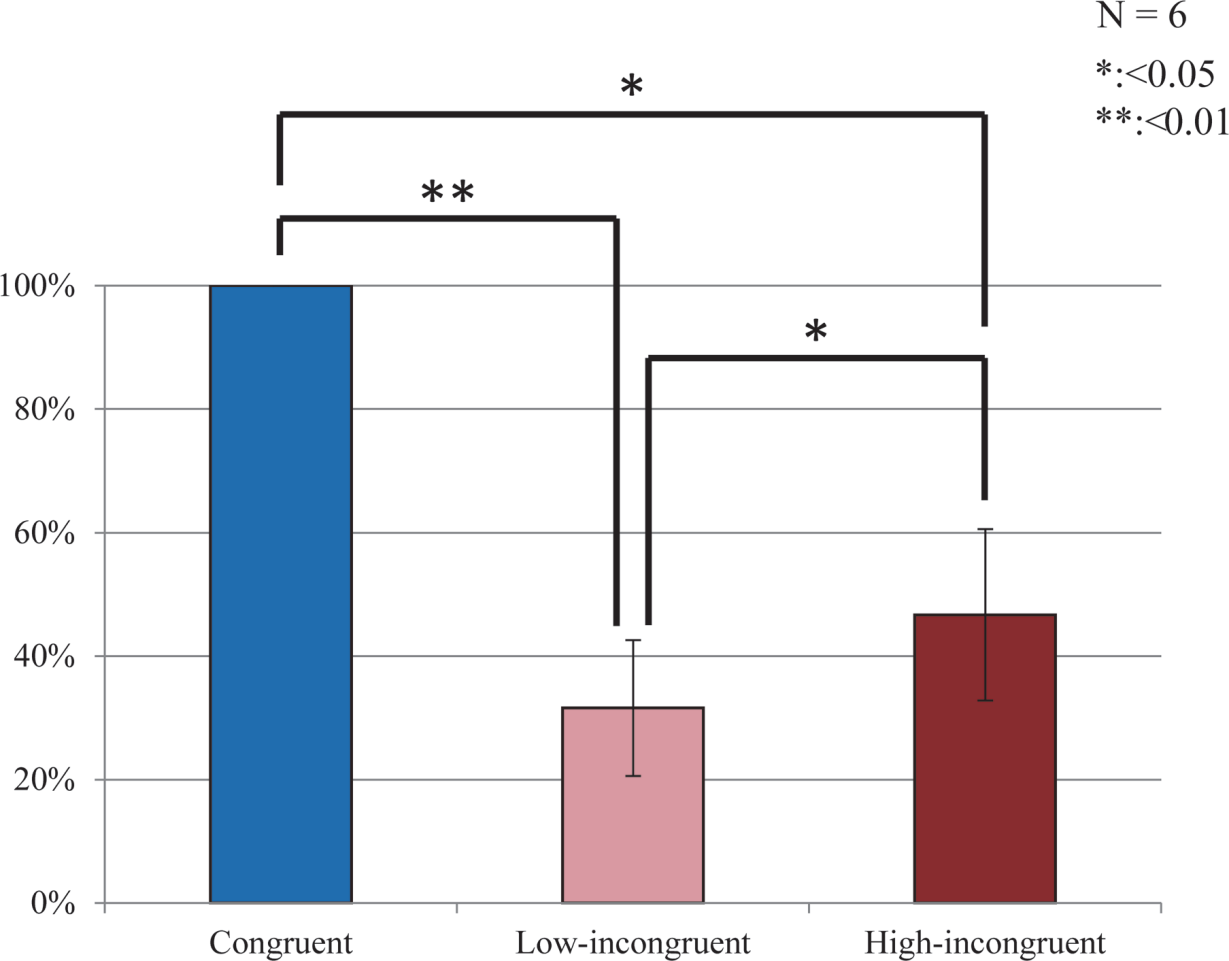
Behavioral results. Average percentages of trials with accurate recognition of target auditory information were 100% (SE = 0.00%), 31.6% (SE = 11.0%) and 46.7% (SE = 13.9%) in the congruent, low-incongruent and high-incongruent conditions, respectively, A two-tailed paired t-test revealed that all the differences were significant (false-discovery rate correction, p < 0.05). Error bars indicate standard error of the mean.

To investigate the effects of the speaker’s lip movement on patients’ perception of the syllable spoken by the voice, we compared the percentage of correct answers between the congruent and incongruent conditions, averaging the low- and high-incongruent conditions, and between the two incongruent conditions. All comparisons of behavioral data were performed using a two-tailed paired t-test (Fig 2). The obtained p-values were corrected for multiple comparisons across the electrodes (false-discovery rate correction, p < 0.05).

Besides the conditions described above, patients also performed the auditory-only condition in which only the voice (i.e., “pa,” “ka,” or “ta”) was presented with the speaker’s mouth masked. We asked patients to answer the voice they heard, similarly to the aforementioned audiovisual conditions, in order to analyze effects of visual information on selective auditory processing. The auditory-only condition consisted of 30 to 60 trials per patient (Fig 1).

### Data acquisition

Each patient underwent electrode implantation approximately four weeks before resection surgery. The patients had grid- and strip-type subdural electrodes placed over the left lateral frontal, temporal, and occipital regions. Based on previous findings on the involvement of the lateral surface of the left hemisphere in audiovisual processing [4,17,30], we focused solely on ECoG electrodes in these regions. The grid electrodes consisted of silastic sheets with 60 platinum electrodes of 1.5 mm diameter with 5 mm spacing (Unique Medical, Tokyo, Japan). Electrode locations were identified by post-implantation computed tomography (CT) registered to pre-implantation magnetic resonance imaging (MRI) based on the mutual information method using Dr.-View/Linux (Asahi-Kasei Information Systems, Tokyo, Japan) [31]. The three-dimensional brain surface was reconstructed using Real INTAGE (Cybernet Systems, Ltd., Tokyo, Japan). There were no epileptic seizure events during or in the 24 h before ECoG recordings. ECoG data were sampled at 2000 Hz using a multi-channel EEG system (EEG 1200, Nihon Koden Corp., Tokyo, Japan). The band-pass filter for the data acquisition was set to 0.08–600 Hz. Event triggers that indicated movie clip onset and voice onset were recorded. A reference electrode was placed on the inner surface of the dura mater over the right parietal lobe.

### Data processing and analysis

All ECoG data were analyzed using a custom script written in Matlab R2012b. First, we excluded the electrodes with continuous extrinsic noise from further analyses. Then, we rejected the data epochs with singular noise and epileptiform spikes. The average number of investigated electrodes per patient was 176 (SE = 2.33).

**Fig 3:**
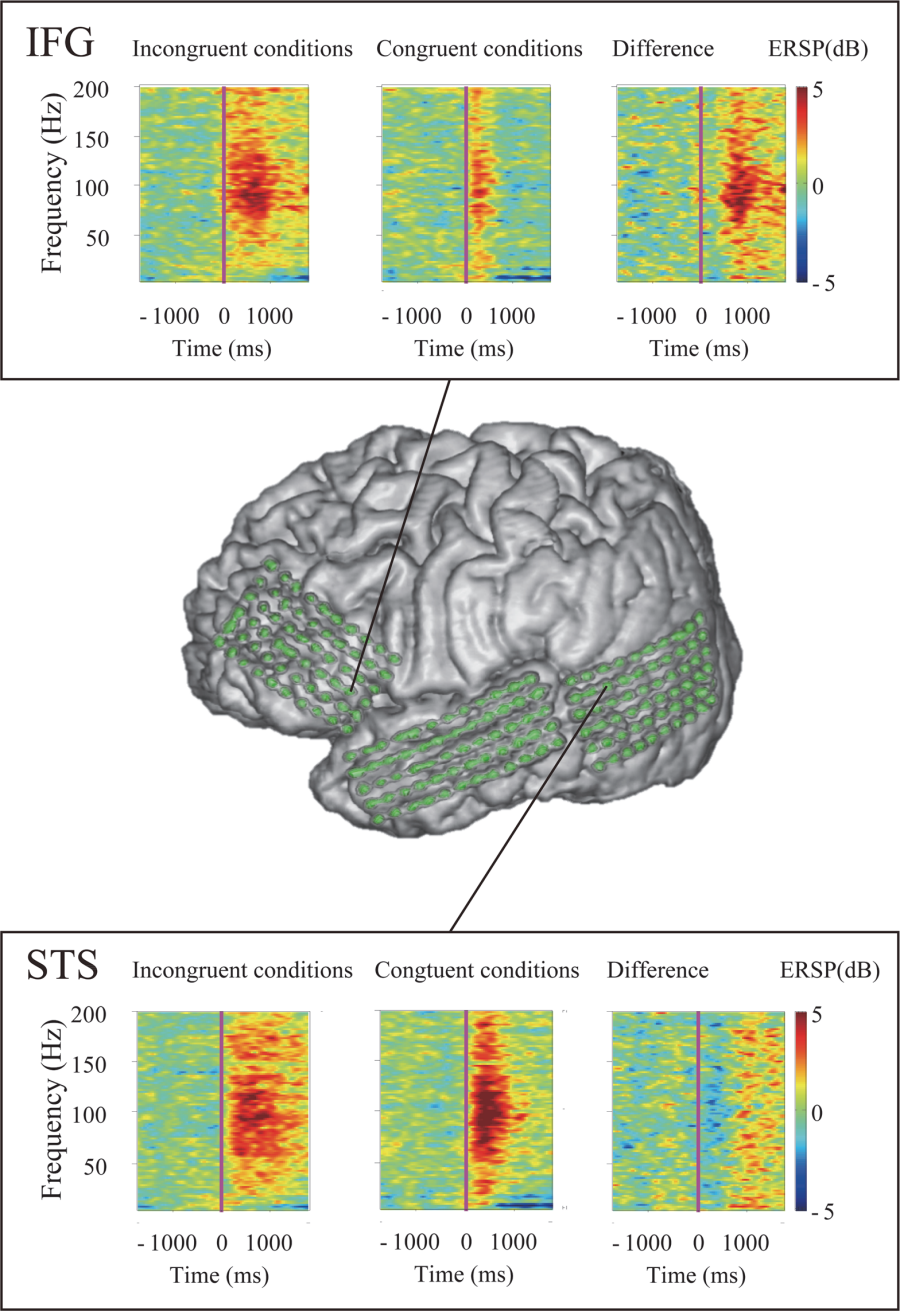
A representative result of time-frequency analysis (patient 3). The result indicates that the neural activity for processing audiovisual mismatch appeared mainly in the high gamma band (70–150Hz).

To obtain the event-related spectral perturbation (ERSP) of each epoch, we used wavelet analysis implemented in the EEGLAB toolbox [32]. ERSP means the event-related changes in amplitudes in oscillatory brain activity for each frequency [33,34]. The ECoG signals were convolved with Hanning-windowed sinusoidal wavelets. The number of wavelet cycles increased with frequency (starting at three cycles in 6 Hz) for optimal time-frequency domain. We analyzed epochs from 1500 ms before voice onset to 1500 ms after voice onset in 5–200 Hz frequency range. We used the default parameters for Morlet wavelet cycles in EEGLAB. The ERSP time-frequency matrices were expressed as percent changes from averaged baseline activities between 1350 to 1050 ms before voice onset, during which a fixation point was presented at the center of the screen. We illustrated the ERSP time-frequency matrices of the congruent, incongruent and difference conditions, which was made by subtracting the averaged spectrograms in the congruent condition from the averaged spectrograms in the incongruent conditions (Fig 3). As represented by Fig 3, the spectrogram of each patient showed a remarkable event-related change in the high gamma band, whereas we found little power changes in other frequency bands.

**Fig 4:**
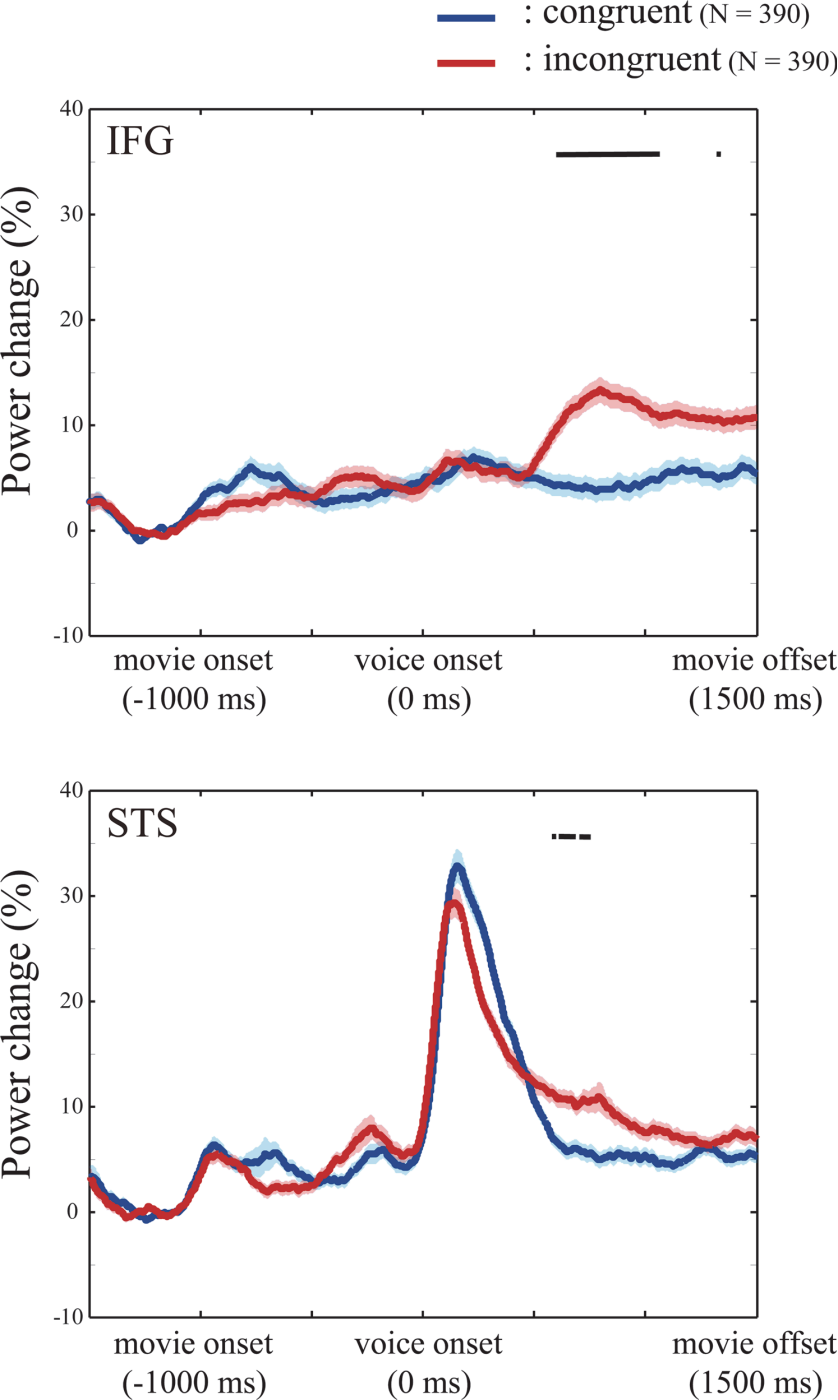
Temporal dynamics of high gamma activity (HGA). The percentage changes of HGA in the inferior frontal gyrus and superior temporal sulcus are calculated in the congruent (blue) and incongruent (red) conditions, respectively. Shading means the standard error of mean. N represents the number of trials. The horizontal black bar indicates the epoch in which the increase in HGA was greater in the incongruent conditions than in the congruent condition (p < 0.0001). The results suggest that audiovisual mismatch increased HGA in later period (500–1000 ms after voice onset).

Then, a finite impulse response filter was first applied to the ECoG data from each electrode of each patient to extract the signals containing high gamma band activity ranging from 70 to 150 Hz [35]. Hilbert transformation was performed on the filtered data and power estimates were computed using the absolute value of these complex numbers [36]. We smoothed the power with a 100-ms boxcar kernel [37] before epoching to illustrate the ERSP in the high gamma band, but did not smooth the power used for statistical analysis. To illustrate temporal dynamics of HGA in the IFG and STS, the percentage changes of HGA in the IFG and STS electrodes (S2 Fig) from the baseline period are calculated on a single trial basis across all patients (Fig 4). We decided that the target periods were from 0 to 500 ms and from 500 to 1000 ms after voice onset, and the baseline was same as the ERSP calculation. We calculated the percentage change in HGA from the baseline to each target period. Hereafter, when we mention increases in HGA, it means the percentage change of HGA from the baseline period.

We compared increases in HGA between the congruent and incongruent conditions to investigate the neuronal activity for audiovisual mismatch processing. A one-tailed t-test was performed for each electrode to examine whether the HGA increase was larger in the incongruent conditions than in the congruent condition or not. For each patient, the obtained p-values were corrected for multiple comparisons across the electrodes (false-discovery rate correction, p < 0.05). In the later target period from 500 to 1000 ms after voice onset, we observed quite a large number of electrodes with significantly larger HGA in the incongruent conditions than in the congruent condition, in contrast with few electrodes with significant HGA in the earlier target period from 0 to 500 ms (see Result section for details). On the basis of these results, we concentrated only on the later period (500–1000 ms) in subsequent analyses.

Three different syllables (i.e., “pa,” “ka,” or “ta”) were used in the congruent condition, therefore we compared the HGA increase across these syllables. There were four electrodes at which the increase or decrease in activity differed across syllables (p < 0.05, one-way ANOVA, false-discovery-rate corrected) [37]. We excluded data from these electrodes from all further analyses.

### Visualization of electrodes on a template brain and selection of electrodes

**Fig 5:**
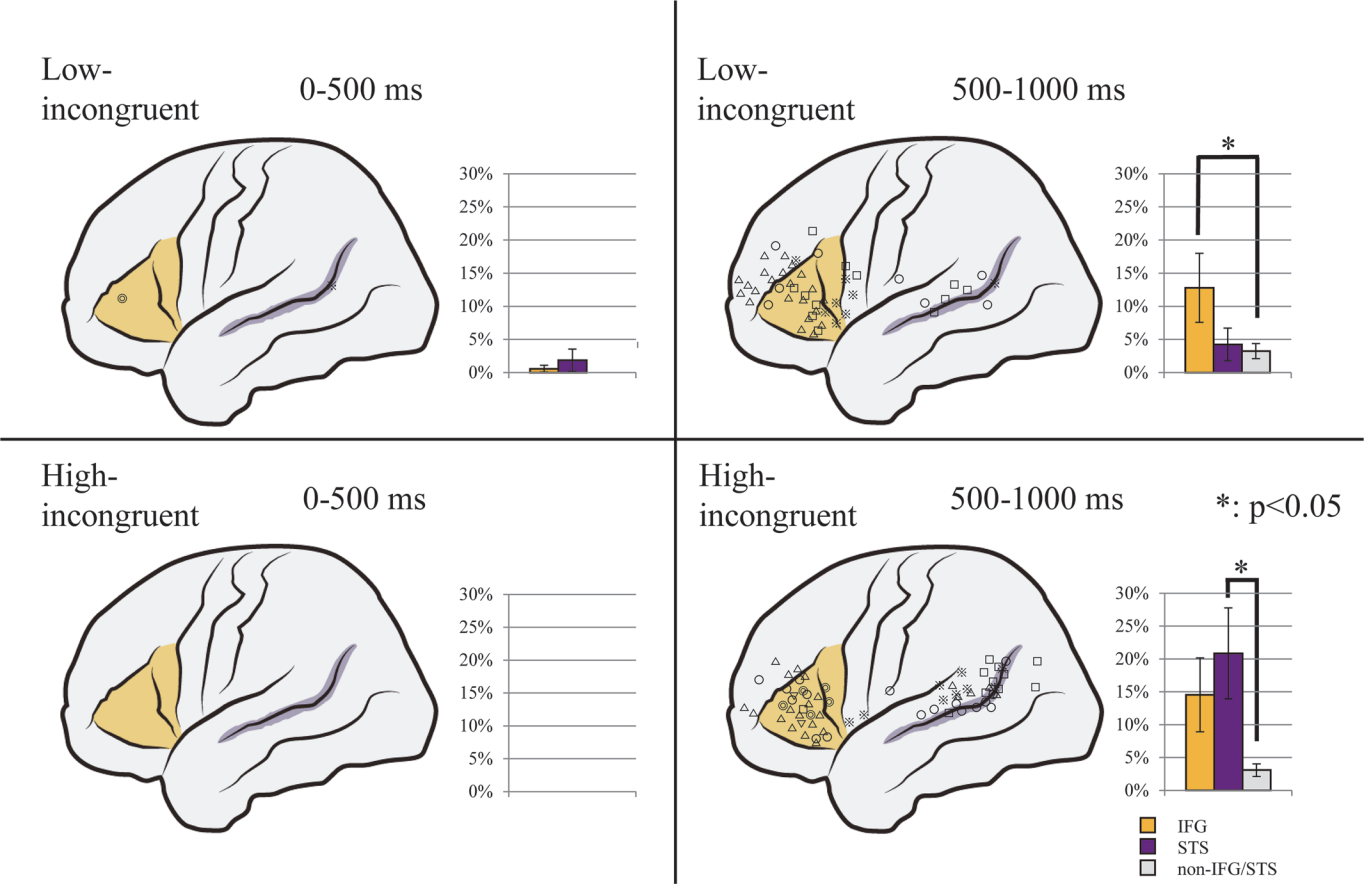
Distribution maps of the high gamma activity (HGA). All the electrodes with a significantly greater HGA increase in the incongruent condition than in the congruent condition are shown on a template brain in the low-incongruent (upper) and high-incongruent (lower) conditions. The shapes of the electrode markers indicate individual patients. The bar charts beside the template brain image show the average percentage of electrodes that showed a significant HGA increase in the inferior frontal gyrus (IFG; orange), superior temporal sulcus (STS; purple), and other (gray) regions respectively. In the early period, in both the low- and high-incongruent conditions, only a few of the 1054 electrodes exhibited a significant HGA increase (left). In the later period, more electrodes showed a significant HGA increase. The increase was localized in the IFG in the low-incongruent condition (p = 0.0462; right upper) and in the STS in the high-incongruent condition (p = 0.0254; right lower). Error bars indicate standard error of the mean.

To elucidate the brain regions involved in audiovisual mismatch processing, we compared HGA increases in the incongruent conditions than those in the congruent condition in each electrode applying an unpaired one-tailed t-test with an FDR correction on all trials, and mapped the electrodes that showed a significantly greater HGA increase in the incongruent conditions than in the congruent condition onto a template brain (Fig 5). We next selected the electrodes in the IFG and STS for later comparison. An electrode was regarded as an IFG electrode if more than half of the electrode was located over the IFG. The STS was defined as the part of the STS that is posterior to the foot of a line perpendicular from the intersection between the Sylvian fissure and central sulcus. The electrodes were not actually placed into sulci; therefore we selected electrodes whose center was located within 1.5 mm of the lateral surface of the STS. The average number of the electrodes in the IFG and STS was 28.7 (SE = 1.87) and 10.0 (SE = 1.43), respectively (S2 Fig).

### Association between HGA and speech perception

We compared the HGA increase between success and error trials in the incongruent conditions to analyze the association of incongruent voice-induced HGA with accurate perception of the syllable spoken by the voice. Here, we classified the behavior not into the McGurk (fusion) or non-McGurk (non-fusion), but into the correct or incorrect answer (success or error trial). For each electrode, we calculated the average and standard deviation increase in HGA across all patients and divided the difference between this average and the increase in HGA in a trial by the standard deviation. This procedure resulted in a standard HGA score for each trial, eliminating individual difference. A two-tailed paired t-test was used to compare the average standard scores of success and error trials across all electrodes of all patients. This was performed separately for the IFG and STS.

## Results

### Behavioral results

We compared the accuracy in judging the syllable spoken by a voice between conditions of different audiovisual congruency to examine the effects of visual information on auditory processing. The percentage of trials in which the patient correctly identified the syllable spoken by the voice was 100% (SE = 0.00%), 31.6% (SE = 11.0%) and 46.7% (SE = 13.9%) in the congruent, low-incongruent and high-incongruent conditions, respectively, and all the differences were significant (Fig 2). It was confirmed that not only the classic McGurk condition, that is the combination of auditory “pa” and visual “ka,” but also the non-classic McGurk condition, or auditory “pa” and visual “su,” are related with decreased accuracy in auditory recognition. In the auditory-only condition (and still-face condition), every patient gave correct answers in all trials (SE = 0.00%). These results suggested that the patients paid enough attention during the recording regardless of task conditions. This indicates that an incongruent lip movement of speaker prevented patients from accurately processing the spoken syllable and that this effect was dependent on the degree of audiovisual incongruence.

### Spectral power of the high gamma band

To detect the brain regions that processed mismatch between auditory and visual inputs, we compared the increase in HGA between trials with and without audiovisual mismatch. HGA increased more in trials in the incongruent conditions than in the congruent condition, particularly in the epoch from 500 to 1000 ms after voice onset (Fig 4).

In the target period from 0 to 500 ms after voice onset, the average percentage of electrodes with larger HGA in the *low*-incongruent condition than in the congruent condition was 0.575% (SE = 0.525%), 1.85% (SE = 1.69%) and 0.00% (SE = 0.00%) in the IFG, STS, and the other regions (non-IFG/STS regions), respectively, and the average percentage of electrodes with larger HGA in the *high*-incongruent condition was 0.00% (SE = 0.00%) in all regions. The increase in HGA was more prevalent in the later period, from 500 to 1000 ms after voice onset, where the average percentage of electrodes with larger HGA in the *low*-incongruent condition than in the congruent condition was 12.8% (SE = 5.22%), 4.23% (SE = 2.47%) and 3.24% (SE = 1.13%) in the IFG, STS, and non-IFG/STS regions, respectively, and the average percentage of electrodes with larger HGA in the *high*-incongruent condition was 14.5% (SE = 5.62%), 20.8% (SE = 6.92%) and 3.07% (SE = 0.950%), respectively. Since we focused on higher-order processing (i.e., audiovisual mismatch processing), the target epoch might be later 500 to 1000 ms than simple voice perception [38], although previous ECoG study also revealed high-frequency brain activity in similarly later period [39–41].

Mapping the electrodes that exhibited a significant difference between incongruent and congruent trials onto a template brain revealed that the HGA increase was localized in the IFG and STS (Fig 5). A one tailed t-test showed that in the low-incongruent condition, the percentage of electrodes that exhibited the increase in HGA in the incongruent condition compared to the congruent condition was more likely to be observed in the IFG than non-IFG/STS regions (p = 0.0462), and in the high-incongruent condition, the percentage of electrodes that exhibited the increase in HGA was larger in the STS than in the non-IFG/STS regions (p = 0.0254; Fig 5).

**Fig 6:**
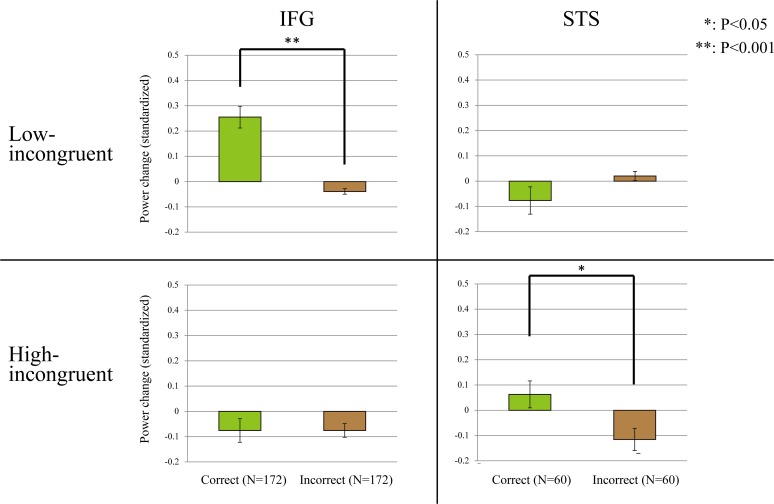
Relation between high gamma activity (HGA) and voice perception. Across all electrodes of all patients, average standard increases in HGA were statistically compared between success and error trials. In the inferior frontal gyrus (IFG), the voice-induced standardized increase in HGA was significantly higher in correct trials than in incorrect trials in the low-incongruent condition (p = 4.76E-9, two-tailed paired t-test). In the superior temporal sulcus (STS), the voice-induced standardized increase in HGA was significantly higher in correct trials than in incorrect trials in the high-incongruent condition (p = 0.0291, two-tailed paired t-test). N represents the number of electrodes in the IFG and STS. The y axis shows the average percentage of standardized power change in HGA. Error bars indicate standard error of the mean.

We also performed behavior-based analysis to examine whether the HGA increase in the IFG and STS was associated with degrees of audiovisual mismatch. We compared the standardized increase in HGA between success and error trials in the high and low incongruent conditions. In the high-incongruent condition, there was no significant difference in the HGA standardized increase in the IFG between success and error trials (p = 0.999, two-tailed paired t-test), but the HGA standardized increase in the STS was significantly larger in success trials than in error trials (p = 0.0291, two-tailed paired t-test). The opposite pattern was observed for the low-incongruent condition, where there was no significant difference in the HGA standardized increase in the STS between success and error trials (p = 0.132, two-tailed paired t-test), but the HGA standardized increase in the IFG was larger in success trials than in error trials (p = 4.76E-9, two-tailed paired t-test; Fig 6). These results suggest a dissociated role of the STS and IFG in processing audiovisual information with large and small discrepancy, respectively.

## Discussion

In the present study we investigated whether the IFG and STS were involved in top-down and bottom-up processing of target auditory information from audiovisual inputs. For this purpose, we recorded the HGA in these regions in the left hemisphere while patients judged the syllable spoken by a voice while viewing lip movements of the speaker. The results showed that the IFG exhibited stronger HGA when the speaker’s voice was judged accurately only if the auditory information was not clearly different from that predicted from the speaker’s lip movement. By contrast, the STS exhibited stronger HGA when the speaker’s voice was judged accurately only if there was a large discrepancy between the spoken syllable and the lip movement (Fig 6). Targets of a small and large discrepancy to distractors are processed in a top-down and bottom-up manner, respectively [11–13]; therefore, these results may suggest that the roles of the IFG and STS are top-down and bottom-up processing of target auditory information from among audiovisual inputs, respectively. In other words, the neural basis of auditory processing changes dynamically in accordance with the degree of incongruence between auditory and visual information.

These findings further our understanding of the role of the STS and IFG in audiovisual processing. The STS is an auditory-visual association area located between the auditory and visual cortices, and non-human primate and human studies have reported its involvement in audiovisual processing [42–46]. Specifically, STS neurons in the monkey were responsive to both auditory and visual stimuli [47–50]. The human STS showed stronger activation when subjects processed audiovisual information than when they processed unimodal input [51–54], and STS activity was more enhanced if there was a discrepancy between auditory and visual information (e.g., a voice speaking the syllable “pa” and lip movements corresponding to the syllable “ka”) than if there was no discrepancy between auditory and visual information [55]. These findings suggest that the STS is important for processing audiovisual mismatch [56,57]. The results of this study revealed that HGA in the STS was associated with auditory processing performance if there was a clear difference between the auditory and visual inputs, but not if the difference between auditory and visual inputs was small. Considering that a highly discrepant target is processed in a sensory-driven manner [11–13], the STS would play a key role in bottom-up detection of the audiovisual mismatch, not audiovisual mismatch processing generally. Taken together with the findings from the unimodal literature [18], this indicates that the superior temporal region is involved in bottom-up detection of unimodal and cross-modal mismatch. Here, we need to note that congruent “su” or visual-only “su” was not presented in this study. Therefore, the effect of visual “su” was not clear in the high-incongruent condition. In other words, this study left a possibility that “su” was a more powerful stimulus than “pa” and the significant response in the STS was due to this factor, rather than congruence or incongruence.

Previous studies have also identified involvement of the IFG in audiovisual processing [17,58]. Brain activity in the IFG was higher when processing audiovisual stimuli than when processing unimodal stimuli [30,59,60], and incongruent audiovisual inputs further increased IFG activity [16,30,61,62]. These properties are similar to those of the STS, and do not show the functional difference between the IFG and STS. However, in this study, we showed that accurate processing of target auditory information induced a larger amount of HGA in the IFG only if the target stimulus resembled the auditory information predicted from the visual input (Fig 6). Considering the properties of processing for a less discrepant target, a primary role of the IFG in audiovisual processing would be top-down detection of audiovisual mismatch.

Evidence from previous studies have suggested that gamma-band activity is involved in bottom-up processing only, and that top-down information is processed through beta-band oscillations [63–65]. Based on these studies, it is possible that the activity shown in the STS and IFG simply represent different points along the same processing stream to detect incongruencies. In other studies, it is reported that the pre-stimulus beta-band activity plays an important role in subjects’ perception in the McGurk paradigm [66]. Thus, beta-band activity or pre-stimulus period activity, on which we did not focus in this study, may have more impact on our recognition. To address this issue we must conduct the temporal analysis, connectivity study and cortical stimulation for the future study.

This study has several other methodological limitations. The HGA responses in this study were later (500–1000 ms after auditory onset) than the time window (60–200 ms) of the audiovisual syllables perception shown by previous researches [67]. Such a difference in latencies seems to support our view that the HGA responses in this study are associated with mismatch detection or judgment, not a simple perception. The temporal window used in this analysis was, however, too long to lead to a reliable speculation on the origin of the activities. In addition, the speculation on the top-down and bottom-up processing in this study lacks a detailed temporal analysis which could discriminate sequential involvements of distributed functional cortical areas. We need to address these issues in the future study.

Finally, since our study was performed for patients with intractable epilepsy, we must consider this pathological factor. Many researches were conducted using the data from patients with intractable epilepsy, and effects of pathological brain lesions and uses of anti-epileptic drugs have been discussed [68]. In our cases, Patient 1 and 6 had epileptic foci close to the STS. They showed the lowest and highest cognitive performance in our tasks, respectively (S3 Fig). This might have influenced the results to some extent. In addition, since we focused on the higher-order processing, we need to evaluate whether the patients carry out the tasks as well as healthy subjects do. The behavioral results of patients in this study were similar with those of healthy subjects (S1 Fig). Our data, therefore, seems reliable, although careful consideration is needed to generalize the results of this study.

## Conclusions

This study showed for the first time that the neural basis of processing target auditory information from audiovisual inputs changed dynamically between the IFG and STS, according to the target-distractor discrepancy. This finding was obtained using ECoG to measure HGA, which is correlated with neuronal firing, in extensive lateral surface regions with high temporal and spatial resolution. It is difficult to achieve all measurements from other brain imaging techniques such as functional magnetic resonance imaging (fMRI); however, ECoG is not able to assess activity in areas where electrodes are not placed, such as intrasulcus activity [69]. Therefore, further research is required to combine other brain imaging techniques with ECoG, and this will provide a more comprehensive understanding of the neural basis for selective processing of auditory information from audiovisual inputs.

## Supporting Information

S1 Dataset Data Standardized HGA in the IFG and STS. (PDF)

S1 Fig Behavioral results of ten healthy subjects. Average percentages of trials with accurate recognition of target auditory information were 99.3% (SE = 0.4%), 18.5% (SE = 8.2%), and 56% (SE = 10.7%) in the congruent, low-incongruent, and high-incongruent conditions, respectively. A two-tailed paired t-test revealed that all the differences were significant (false-discovery rate correction, p < 0.05). This performance profile was similar with those of patients in the study. Error bars indicate standard error of the mean.
(TIF)

S2 Fig The location of electrodes. The total number of electrodes in this study was 1054, which are shown on a template brain. The inferior frontal gyrus (IFG; pars opercularis and triangularis) and the posterior part of superior temporal sulcus (STS) had 172 (yellow) and 60 (purple) electrodes respectively; and there were 822 (green) electrodes in the non-IFG/STS regions.
(TIF)

S3 Fig Behavioral results of six patients. Since the presented voice was “pa” in both conditions, we judged that patients could process the voice adequately if they chose “pa.”
(TIF)

S4 Fig Distribution maps of the high gamma activity in uni/multi-sensory inputs. The electrodes which showed a significantly greater HGA increase from baseline period (1350–1050 ms before voice onset) to the target period (0–1000 ms after voice onset) in the auditory-only (left), congruent (middle), and incongruent (right) conditions (Bonferroni correction across all electrodes of each patient, p < 0.05). The HGA increases were localized in the superior temporal sulcus in all conditions (p = 0.0036, 0.012, and 0.034 for auditory-only, congruent, and incongruent conditions, respectively), but not in the inferior frontal gyrus. The shapes of the electrode markers indicate individual patients.
(TIF)
