# Supplementary material for: Dissociated Roles of the Inferior Frontal Gyrus and Superior Temporal Sulcus in Audiovisual Processing: Top-Down and Bottom-Up Mismatch Detection
Source: PLoS One. 2015 Mar 30;10(3):e0122580. doi: 10.1371/journal.pone.0122580 (PMC4379108; doi:10.1371/journal.pone.0122580)
Supplement: S1 Dataset — (PDF) [file pone.0122580.s001.pdf]

HGA standardized increase for each electrode

inferior frontal gyrus

The low-incongruent condition		The high-incongruent condition	
Correct	Incorrect	Correct	Incorrect
0.053409	-0.03561	-0.07174	0.167382
-0.06874	0.045828	-0.0961	0.224222
-0.08482	0.056543	-0.13729	0.320349
0.148185	-0.09879	0.020443	-0.0477
0.109593	-0.07306	-0.02186	0.051019
-0.1468	0.097867	0.241508	-0.56352
-0.12725	0.084833	-0.13911	0.32458
0.028204	-0.0188	0.029157	-0.06803
0.24756	-0.16504	-0.19434	0.45345
0.2305	-0.15367	-0.11592	0.27048
-0.00376	0.002508	-0.04722	0.110182
-0.02769	0.018461	-0.11392	0.265809
0.187983	-0.12532	0.001766	-0.00412
-0.08124	0.05416	0.007937	-0.01852
0.282416	-0.18828	-0.09836	0.229497
0.218418	-0.14561	-0.16963	0.395816
-0.03826	0.025507	-0.05247	0.122437
-0.09107	0.060716	-0.12907	0.301157
0.335144	-0.22343	0.017156	-0.04003
0.099261	-0.06617	-0.01535	0.035821
1.312771	-0.14586	0.811273	-0.09014
-0.00103	0.000114	-0.01306	0.00145
0.480327	-0.05337	-0.71449	0.079388
1.305185	-0.14502	1.56877	-0.17431
0.853966	-0.09489	0.709311	-0.07881
0.799534	-0.08884	0.556633	-0.06185
0.888286	-0.0987	-0.38078	0.042309
0.69428	-0.07714	0.876209	-0.09736
1.662913	-0.18477	0.990285	-0.11003
1.843701	-0.20486	0.971909	-0.10799
1.216606	-0.13518	0.516691	-0.05741
-0.01049	0.001166	-0.58566	0.065073
0.709605	-0.07884	-0.25653	0.028503
0.110827	-0.01231	0.596069	-0.06623
-0.1753	0.019478	-0.61747	0.068608
0.571819	-0.06353	-0.07381	0.008201
-0.02238	0.002486	-1.93076	0.214529
-0.18479	0.020533	-1.08034	0.120038
-0.14588	0.016208	-0.93714	0.104127
0.291336	-0.03237	0.851332	-0.09459
0.392322	-0.04359	2.446763	-0.27186
0.136725	-0.01519	-0.77263	0.085848
-0.21876	0.024307	-0.37168	0.041298
-0.63368	0.070408	1.218423	-0.13538
-0.35494	0.039437	-0.51363	0.057071
0.962639	-0.10696	-0.00134	0.000148
0.66991	-0.07443	-0.64217	0.071352
0.778757	-0.08653	0.371144	-0.04124
0.308411	-0.03427	-0.85224	0.094693
-1.47926	0.077856	-1.49316	0.165907
-0.3628	0.019095	-1.69696	0.188551
1.329315	-0.06996	0.121226	-0.01347
1.067808	-0.0562	-1.00721	0.111912
1.885921	-0.09926	-1.61648	0.179609

0.137746	-0.00725	-0.95132	0.105702
0.324711	-0.01709	-0.34784	0.038649
0.731951	-0.03852	1.018362	-0.11315
0.845561	-0.0445	-1.29184	0.143538
2.619485	-0.13787	1.70895	-0.18988
0.227423	-0.01197	-0.3191	0.035455
0.970665	-0.05109	-0.02716	0.003018
-0.04359	0.002294	-0.03731	0.004145
0.849928	-0.04473	-0.89973	0.099969
0.197633	-0.0104	-0.79249	0.088054
-0.34774	0.018302	-0.40183	0.044648
1.147578	-0.0604	-1.28143	0.142382
0.932479	-0.04908	0.499185	-0.05546
0.771426	-0.0406	-0.51491	0.057212
-0.01436	0.000755	1.654967	-0.18388
-1.02316	0.053851	-1.64185	0.182428
-0.30443	0.016022	-1.57258	0.17473
-0.84954	0.044712	-0.90942	0.101047
-0.34379	0.018094	-0.73649	0.081833
0.382178	-0.02011	-0.72858	0.080953
1.267744	-0.06672	-1.91276	0.212528
0.579961	-0.03052	-2.04394	0.227105
-0.0374	0.077672	0.08493	-0.76437
0.138155	-0.28694	0.079899	-0.71909
0.174155	-0.3617	0.008777	-0.079
0.154789	-0.32149	0.151971	-1.36773
0.098743	-0.20508	-0.13712	1.234045
0.146612	-0.3045	-0.0047	0.042276
0.257677	-0.53517	0.016545	-0.14891
-0.01901	0.039479	0.175142	-1.57628
0.031067	-0.06452	0.080768	-0.72691
0.055399	-0.11506	0.024643	-0.22178
-0.022	0.04569	0.021388	-0.19249
0.164097	-0.34082	0.041611	-0.3745
0.211694	-0.43967	0.087208	-0.78487
0.257045	-0.53386	0.014939	-0.13445
0.043513	-0.09037	0.001877	-0.01689
0.088155	-0.18309	0.089446	-0.80501
0.155642	-0.32325	0.024783	-0.22305
0.14047	-0.29175	0.102393	-0.92155
0.119872	-0.24897	0.080714	-0.72643
0.039742	-0.08254	0.063477	-0.57128
0.128648	-0.26719	-0.02661	0.239452
0.048649	-0.10104	0.038674	-0.34806
0.071096	-0.14767	-0.09775	0.879717
0.150144	-0.31184	-0.02087	0.187873
0.194841	-0.40467	0.097508	-0.87757
0.154087	-0.32003	0.018524	-0.16671
-0.05285	0.10976	0.040791	-0.36711
0.015957	-0.03314	-0.01062	0.095604
0.0661	-0.13729	-0.05073	0.456557
0.115187	-0.23923	0.054921	-0.49428
-0.25041	0.520086	-0.00707	0.063591
0.055205	-0.11465	0.074347	-0.66914
-0.00679	0.014102	0.077667	-0.69901
-0.07547	0.156744	0.164666	-1.482
0.08832	-0.00305	-0.27231	0.068079
0.334944	-0.01155	-0.27998	0.069996
0.048565	-0.00167	0.029241	-0.00731

0.049765	-0.00172	-0.1398	0.034951
0.524134	-0.01807	-0.0258	0.006451
1.489041	-0.05135	0.441254	-0.11031
1.27137	-0.04384	0.138756	-0.03469
0.1556	-0.00537	-0.05906	0.014765
0.067873	-0.00234	-0.10574	0.026436
0.190414	-0.00657	-0.03186	0.007965
0.442594	-0.01526	0.031136	-0.00778
0.965706	-0.0333	0.24402	-0.06101
0.257051	-0.00886	-0.0913	0.022825
1.436233	-0.04953	-0.31282	0.078204
0.983777	-0.03392	-0.11376	0.02844
0.483703	-0.01668	0.750857	-0.18771
0.017949	-0.00062	0.674544	-0.16864
0.220517	-0.0076	-0.12355	0.030887
-0.48605	0.01676	-0.32643	0.081608
1.677702	-0.05785	-0.39763	0.099407
-0.14343	0.004946	0.161226	-0.04031
0.252952	-0.00872	0.164283	-0.04107
0.200633	-0.00692	-0.16356	0.04089
0.387669	-0.01337	-0.43676	0.10919
0.039175	-0.00135	0.025799	-0.00645
1.822749	-0.06285	0.00804	-0.00201
0.964266	-0.03325	-0.23576	0.058941
2.154912	-0.07431	-0.10959	0.027399
-0.31836	0.010978	0.558099	-0.13952
-0.00696	0.012243	-0.00576	0.023032
0.008201	-0.01442	-0.03665	0.146583
0.00183	-0.00322	0.048744	-0.19498
0.019764	-0.03476	-0.00811	0.032429
0.01904	-0.03348	0.058994	-0.23597
-0.11991	0.210872	0.171765	-0.68706
-0.13454	0.236597	0.11814	-0.47256
0.058251	-0.10244	0.052345	-0.20938
-0.05322	0.093591	0.010854	-0.04341
-0.00388	0.006823	-0.04077	0.163061
0.05398	-0.09493	0.090682	-0.36273
-0.00588	0.010333	0.109044	-0.43618
-0.20665	0.363427	0.028666	-0.11466
-0.04573	0.080427	0.114518	-0.45807
-0.01088	0.019128	0.011166	-0.04466
-0.08651	0.152144	-0.07584	0.303369
0.027135	-0.04772	0.037696	-0.15078
-0.01867	0.032831	0.090743	-0.36297
-0.03084	0.054238	0.118419	-0.47367
0.04729	-0.08316	0.096148	-0.38459
0.034838	-0.06127	0.087472	-0.34989
-0.10617	0.186717	-0.01811	0.072423
-0.05679	0.099866	-0.02883	0.115303
-0.04242	0.074599	0.122328	-0.48931
0.002528	-0.00445	0.070021	-0.28009
-0.01697	0.029841	-0.10332	0.413291
-0.12687	0.223124	-0.08653	0.346116
-0.11517	0.202549	0.002821	-0.01128
-0.11651	0.204888	0.146285	-0.58514
-0.13073	0.229896	-0.01809	0.072342
-0.14074	0.247507	0.003048	-0.01219
-0.07427	0.130614	-0.11047	0.441869
-0.14982	0.263485	0.155874	-0.6235

superior temporal sulcus

The low-incongruent condition		The high-incongruent condition	
Correct	Incorrect	Correct	Incorrect
0.366323	-0.24421	-0.21719	0.50677
0.226753	-0.15117	-0.30496	0.711583
-0.04072	0.027144	-0.17821	0.415817
0.044266	-0.02951	-0.00696	0.016241
-0.07951	0.053005	0.064175	-0.14974
0.244302	-0.16287	0.089965	-0.20992
-0.25995	0.173296	-0.00564	0.013158
-0.06644	0.044293	0.117597	-0.27439
-0.02884	0.019228	0.001083	-0.00252
-0.13596	0.015107	-0.48708	0.05412
-0.28115	0.031238	-0.52934	0.058815
0.076785	-0.00853	0.30794	-0.03422
-1.33173	0.14797	-1.29218	0.143576
-1.04121	0.11569	-0.30198	0.033552
-0.48423	0.053804	-0.35675	0.039639
-0.47322	0.024906	0.280911	-0.03121
-0.47082	0.02478	-0.34777	0.038642
-0.25582	0.013464	0.228712	-0.02541
0.053609	-0.00282	-0.86872	0.096524
0.971965	-0.05116	1.364903	-0.15166
-1.48896	0.078366	1.436967	-0.15966
0.129227	-0.2684	-0.00438	0.039422
0.053802	-0.11175	0.022408	-0.20167
0.143742	-0.29854	-0.03034	0.273054
0.040953	-0.08505	-0.04288	0.385948
-0.06148	0.12768	-0.08349	0.751446
-0.07608	0.15801	0.028711	-0.2584
-0.06169	0.128121	-0.0119	0.107088
0.054692	-0.11359	0.080475	-0.72427
-0.18878	0.392088	0.063628	-0.57266
0.121359	-0.25205	0.074663	-0.67197
0.05581	-0.11591	0.039501	-0.35551
-0.2386	0.495542	0.064448	-0.58004
-0.02191	0.04551	0.111381	-1.00243
-0.02195	0.045587	0.016282	-0.14654
-1.26097	0.043482	-0.31283	0.078208
-0.52702	0.018173	0.051824	-0.01296
0.224787	-0.00775	0.71692	-0.17923
-0.22398	0.007723	0.553404	-0.13835
0.347451	-0.01198	0.878649	-0.21966
0.729919	-0.02517	0.040596	-0.01015
0.214845	-0.00741	0.743934	-0.18598
0.787446	-0.02715	0.620377	-0.15509
-0.0797	0.002748	0.047539	-0.01188
0.254582	-0.00878	0.068481	-0.01712
-0.02447	0.043027	0.099116	-0.39646
0.017796	-0.0313	0.05023	-0.20092
-0.00048	0.00084	0.129184	-0.51674
-0.01854	0.032606	0.022215	-0.08886
0.000726	-0.00128	-0.03382	0.135291
-0.07986	0.140437	0.111255	-0.44502
-0.0483	0.084934	0.107341	-0.42936

−0.05057	0.088928		−0.00276	0.011044
−0.14031	0.246746		0.164022	−0.65609
−0.02637	0.046376		0.149152	−0.59661
−0.11085	0.194947		−0.0425	0.170018
0.156906	−0.27594		0.087657	−0.35063
−0.03511	0.061738		0.237676	−0.95071
−0.0293	0.051521		−0.00012	0.000499
−0.13776	0.242272		−0.03244	0.129756
