# Supplementary figures and images for: Dissociated Roles of the Inferior Frontal Gyrus and Superior Temporal Sulcus in Audiovisual Processing: Top-Down and Bottom-Up Mismatch Detection
Source: PLoS One. 2015 Mar 30;10(3):e0122580. doi: 10.1371/journal.pone.0122580 (PMC4379108; doi:10.1371/journal.pone.0122580)

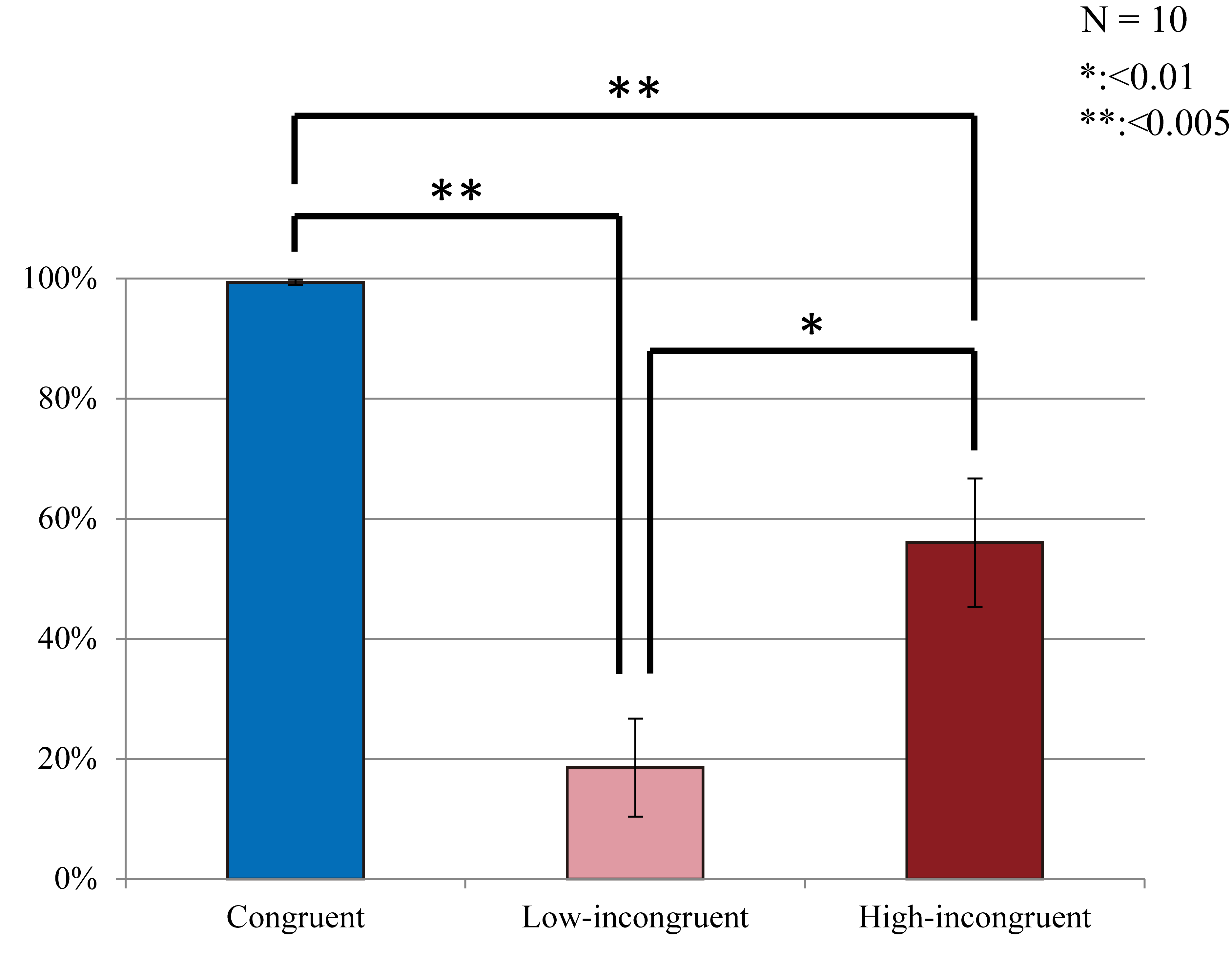

Supplement: S1 Fig — Average percentages of trials with accurate recognition of target auditory information were 99.3% (SE = 0.4%), 18.5% (SE = 8.2%), and 56% (SE = 10.7%) in the congruent, low-incongruent, and high-incongruent conditions, respectively. A two-tailed paired t-test revealed that all the differences were significant (false-discovery rate correction, p < 0.05). This performance profile was similar with those of patients in the study. Error bars indicate standard error of the mean. (TIF) [file pone.0122580.s002.tif]

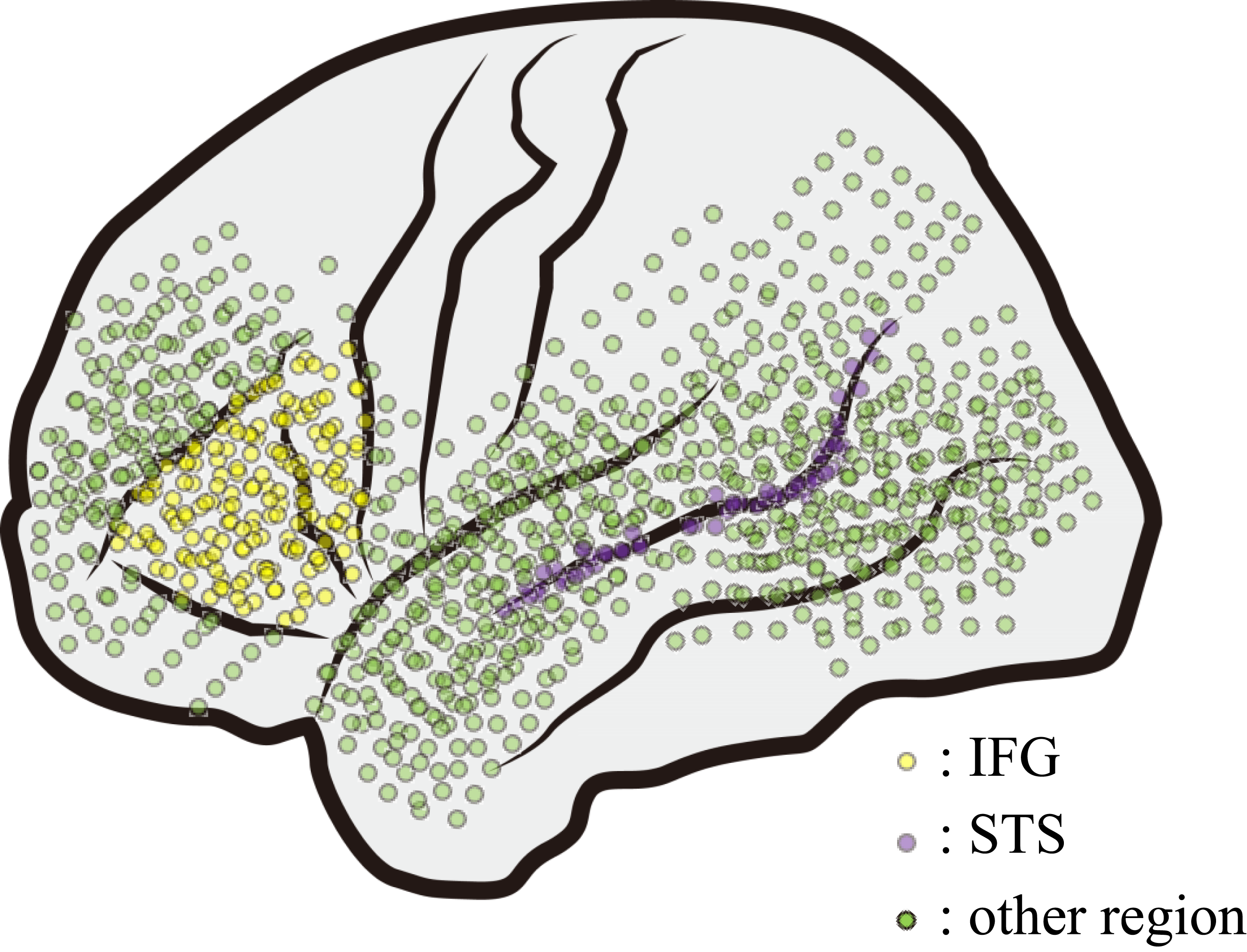

Supplement: S2 Fig — The total number of electrodes in this study was 1054, which are shown on a template brain. The inferior frontal gyrus (IFG; pars opercularis and triangularis) and the posterior part of superior temporal sulcus (STS) had 172 (yellow) and 60 (purple) electrodes respectively; and there were 822 (green) electrodes in the non-IFG/STS regions. (TIF) [file pone.0122580.s003.tif]

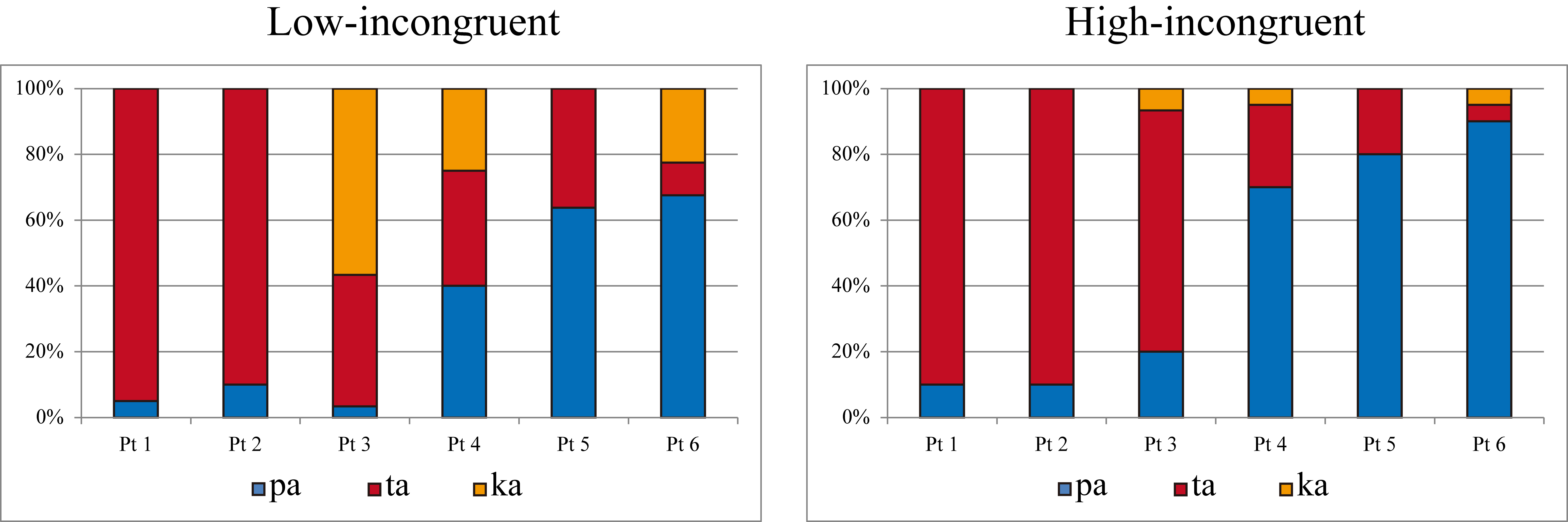

Supplement: S3 Fig — Since the presented voice was “pa” in both conditions, we judged that patients could process the voice adequately if they chose “pa.” (TIF) [file pone.0122580.s004.tif]

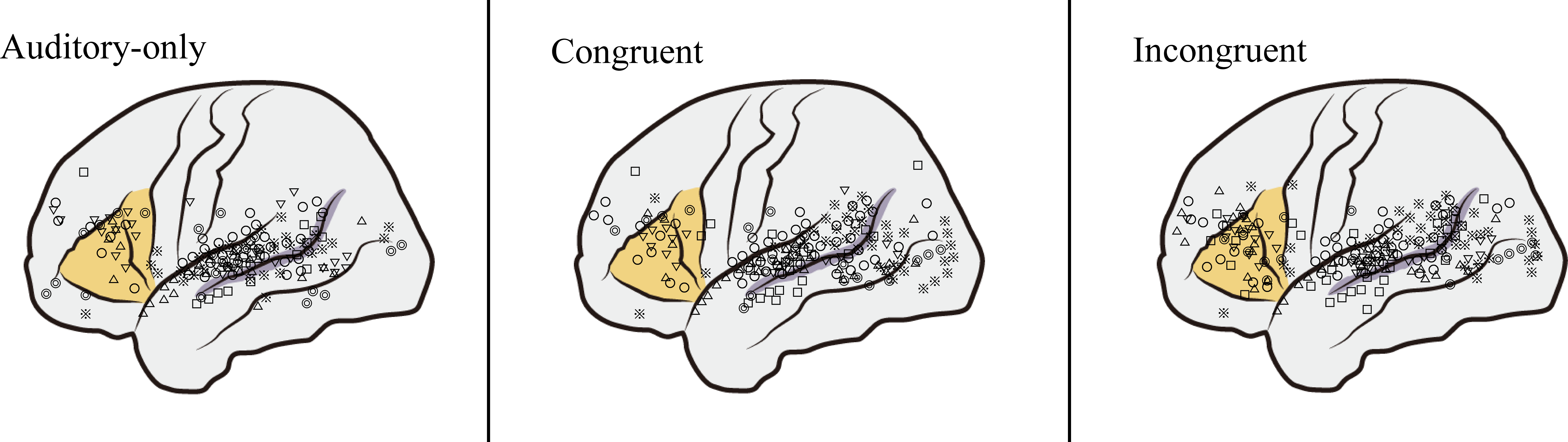

Supplement: S4 Fig — The electrodes which showed a significantly greater HGA increase from baseline period (1350–1050 ms before voice onset) to the target period (0–1000 ms after voice onset) in the auditory-only (left), congruent (middle), and incongruent (right) conditions (Bonferroni correction across all electrodes of each patient, p < 0.05). The HGA increases were localized in the superior temporal sulcus in all conditions (p = 0.0036, 0.012, and 0.034 for auditory-only, congruent, and incongruent conditions, respectively), but not in the inferior frontal gyrus. The shapes of the electrode markers indicate individual patients. (TIF) [file pone.0122580.s005.tif]
